# Perfil de Prescrição de Estatinas e de Níveis Lipêmicos em Ambulatórios de Hospital Terciário Público

**DOI:** 10.36660/abc.20190513

**Published:** 2021-04-08

**Authors:** André Schmidt, Henrique Turin Moreira, Gustavo Jardim Volpe, Vamberto B. Foschini, Thiago Florentino Lascala, Minna Moreira Dias Romano, Marcus Vinícius Simões, José Ernesto dos Santos, Benedito Carlos Maciel, José Antonio Marin

**Affiliations:** 1 Universidade de São Paulo Faculdade de Medicina de Ribeirão Preto Ribeirão PretoSP Brasil Universidade de São Paulo Faculdade de Medicina de Ribeirão Preto (HCFMRP-USP), Ribeirão Preto, SP – Brasil

**Keywords:** Inibidoresde Hidroimetilglutaril CO Redutases, Dislipidemias, Hiperlipoproteinemia Tipo II, Hipercolesterolemia Familiar/terapia, Perfil Lipídico, Hospital Público, Colesterol

## Abstract

**Fundamento:**

O surgimento de nova classe de medicamentos com elevada capacidade de reduzir o LDL-colesterol (LDL-c) renovou o interesse na caracterização da hipercolesterolemia familiar (HF). Pouco se conhece do perfil lipídico de pacientes em atendimento terciário em nosso meio para caracterizar a real ocorrência de HF, que começa a ser suspeitada com níveis de LDL-c acima de 190mg/dL.

**Objetivos:**

O estudo avaliou o perfil lipídico (colesterol total [CT] e LDL-c) de pacientes de hospital público terciário.

**Métodos:**

Estudo retrospectivo de avaliação de prescrições de estatinas e resultados dos lipídios. O nível de significância foi estabelecido em 5%.

**Resultados:**

Em 1 ano, 9.594 indivíduos receberam prescrição ambulatorial de estatinas, 51,5% do gênero feminino, idade média de 63,7±12,9 anos (18 a 100 anos). Trinta e duas especialidades prescreveram estatinas, sendo a cardiologia responsável por 43%. Cerca de 15% das prescrições não tinham dosagem recente de CT, e 1.746 (18,0%) não apresentavam resultado recente de LDL-c. A ocorrência de LDL-c > 130mg/dL e < 190mg/dL ocorreu em 1.643 (17,1%) casos, e 228 (2,4%) apresentaram LDL-c ≥ 190mg/dL dentre os que utilizavam estatinas nas diversas doses. Apenas duas estatinas foram utilizadas: sinvastatina e atorvastatina, e a primeira foi prescrita em 77,6% das receitas.

**Conclusão:**

Nesta coorte transversal de hospital terciário, foi possível verificar que a prescrição de estatinas é disseminada, mas que a obtenção de metas adequadas de CT e LDL-c não é atingida em grande percentual, e que há, possivelmente, significativo contingente de portadores de HF que necessitariam ser investigados por suas implicações prognósticas.

## Introdução

Apesar de metanálise recente sugerir ser de 1:250 a incidência de HF na população geral,^1^ desconhece-se a real prevalência específica de casos com alterações extremas da colesterolemia em serviços públicos ambulatoriais terciários de nosso país. Esses serviços concentram, em geral, os casos com mais comorbidades e maior gravidade clínica.

Alguns estudos avaliaram a custo-efetividade da utilização de estatinas pelo Sistema Único de Saude (SUS);^2^^,^^3^ contudo, a aderência ao tratamento foi pouco estudada em grupos seletos (mulheres), atingindo 15,5% em pequena série.^4^ Com a recente incorporação ao arsenal terapêutico de novos medicamentos altamente eficazes no controle de hipercolesterolemia,^5^^,^^6^ ainda que de elevado custo, os hospitais terciários tornaram-se ponto de convergência de pacientes com perfil lipídico muito alterado, visando à sua prescrição pelo sistema público. No entanto, pouco se conhece do perfil lipídico e do tratamento de pacientes em seguimento ambulatorial nessas instituições.

O objetivo deste estudo consiste em relatar o estado atual da prescrição de estatinas em um hospital público de nível terciário e o grau de controle da dislipidemia assim obtido, além de verificar a possível existência de casos sugestivos de HF (LDL-c > 190mg/dL) mesmo em uso de estatinas.

### Material e métodos

Trata-se de estudo de coorte transversal. Uma coleta sistematizada foi obtida no prontuário eletrônico da Instituição cotejando todos os indivíduos com idade igual ou superior a 18 anos que receberam prescrição ambulatorial de uma estatina no Hospital das Clínicas da Faculdade de Medicina de Ribeirão Preto da Universidade de São Paulo (HCFMRP-USP), hospital terciário de ensino, durante o ano de 2016. Além disso, foram coletadas as dosagens de CT e/ou LDL-c no ano de 2016, posteriormente realizadas ambulatorialmente. Para aqueles que realizaram mais que um exame de lipídios naquele ano, foi considerado o último exame. Idade e gênero foram coletados. Foram também registrados o medicamento prescrito e a dose utilizada, bem como a clínica que a prescreveu quando da realização do último exame. Considerando ser uma busca eletrônica direcionada, não foram coletados dados relativos a comorbidades ou clínicos/antropométricos. Esse estudo foi aprovado pelo Comitê de Ética em Pesquisa local e registrado com o número CAAE 16516819.0.0000.5440.

### Análise estatística

Foi realizada análise descritiva de tais dados, que foram expressos em média e desvio padrão quando a distribuição foi considerada normal pelo teste de Kolmogorov-Smirnov. Variáveis qualitativas foram expressas em porcentagem. Teste de correlação de Pearson foi utilizado para correlacionar os valores de CT e LDL-c. Teste t de Student não pareado foi utilizado para comparação de idades utilizada.

Foi utilizado o programa SPSS v.25 (IBM Corporation, EUA), e o nível de significância foi estabelecido em < 5%.

## Resultados

### Prescrições realizadas

Ao longo de 2016, 9.594 indivíduos receberam prescrição ambulatorial de estatinas na Instituição. Houve discreto predomínio do gênero feminino com 4.942 (51,5%) prescrições, e a idade média foi de 63,7±12,9 anos (18 a 100 anos). Nesses pacientes, foram identificados 8.110 (84,8%) resultados de CT e 7.848 (82,0%) de LDL-c, indicando que 1.484 (15,2%) indivíduos receberam prescrição sem dosagem recente de CT e 1.746 (18,0%) pacientes não apresentavam resultado recente de LDL-c. Ressalta-se que, em todas as especialidades, houve casos de prescrições sem dosagens; no entanto, na cirurgia vascular, isso foi mais saliente, com cerca de 75% das receitas emitidas sem dosagem de LDL-c.

No que tange as 32 especialidades médicas que as prescreveram, a cardiologia foi responsável por 43,5% das prescrições, seguida pela cirurgia vascular com 9,2% e nefrologia com 8,6%. O restante (32%) ficou distribuído em todas as outras especialidades presentes em um hospital terciário público, sendo a nutrologia responsável por apenas 106 (1,1%) receitas ambulatoriais (Tabela 1).

**Tabela 1:** Distribuição das prescrições de estatinas por especialidade médica no ano de 2016

Especialidade	N (%)
Cardiologia	4.160 (43,5)
Cirurgia vascular	1.576 (9,2%)
Nefrologia	819 (8,6)
Neurologia	731 (7,6)
Geriatria	657 (6,9)
Endocrinologia	653 (6,8)
Nutrologia	94 (1,0)
Outras 25 especialidades	1.576 (16,5)
Total	9.567 (100)

### Perfil de lipídios

**Figura 1:**
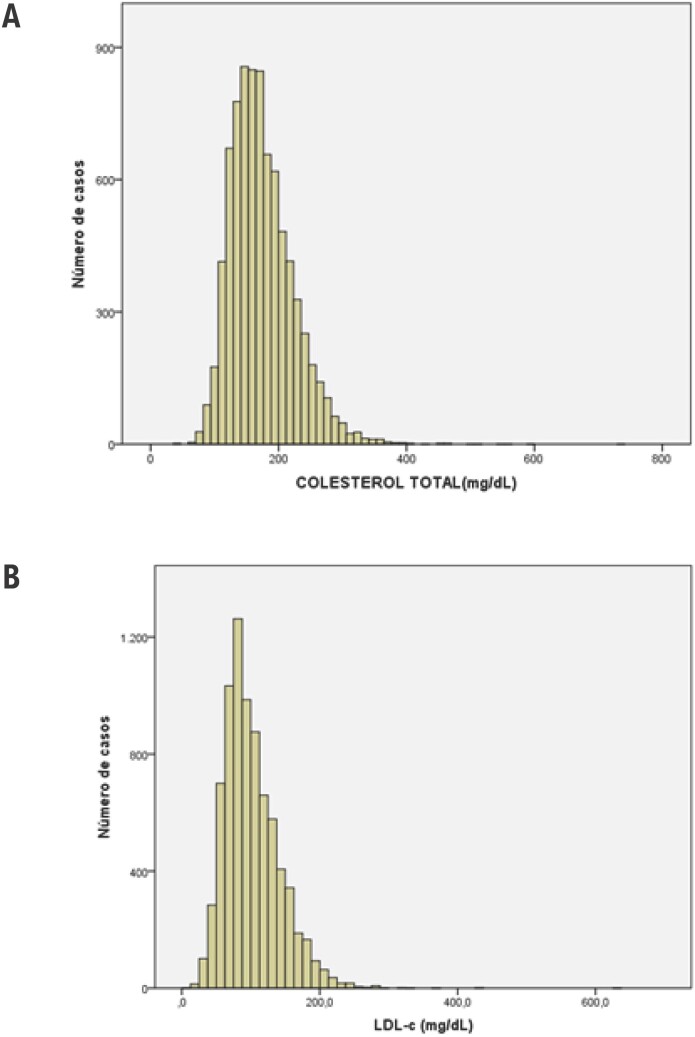
Histogramas de distribuição de frequência dos valores absolutos de colesterol total (A) e LDL-c (B) na amostra de pacientes do Hospital das Clínicas da Faculdade de Medicina de Ribeirão Preto da Universidade de São Paulo (HCFMRP-USP) no ano de 2016.

Colesterol total médio da amostra foi de 174, 4±49,5mg/dL variando entre 40,0 e 739,0mg/dL, enquanto o LDL-c médio foi de 101,1±40,0mg/dL, variando entre 4,0 e 635,0 mg/dL. Uma forte correlação entre os valores de CT e LDL-c foi observada (r=0,94; p<0,001). A distribuição amostral dos valores obtidos para CT e LDL-c está resumida na Figura 1.

Verificou-se que as mulheres apresentaram níveis lipídicos significativamente mais alterados que os homens, tanto para CT (183,3 ± 49,9 *vs.* 164,5±47,0mg/dL; p<0,001) quanto para LDL-c (107,1 ± 40,9 *vs*. 94,3± 7,9mg/dL; p<0,001), ainda que a idade média dos dois gêneros fosse semelhante (63,65±13,56 *vs*. 63,36±12,60; p=0,29).

A ocorrência de LDL-c > 130mg/dL e < 190mg/dL ocorreu em 1.643 (17,1%) indivíduos com prescrição de estatina. Some-se a isso o fato de que 18,2% da amostra total não apresentava dosagem de LDL-c, apesar da prescrição de estatina ambulatorial. Portanto, possivelmente, um considerável percentual de indivíduos apresenta LDL-c acima do preconizado em diretrizes de prevenção primária, apesar do uso de estatinas e sem levar em consideração as entidades nosológicas concomitantes por eles apresentadas.

Finalmente, 228 (2,4%) indivíduos apresentaram LDL-c ≥ 190mg/dL dentre os que utilizavam estatinas nas diversas doses. Dois terços (152) eram do gênero feminino. Este grupo de pacientes apresenta idade média menor que a da amostra total (55±15 anos *vs.* 63±13 p<0,05), podendo este ser um indicador a mais para sugerir a ocorrência de HF neste grupo de pacientes de um hospital terciário.

### Estatinas utilizadas

Por se tratar de hospital público, apenas duas estatinas estão disponíveis para prescrição ambulatorial: sinvastatina e atorvastatina. A primeira foi prescrita em 7.474 (77,6%) receitas. Sinvastatina na dose de 40mg foi prescrita em 3.760 (39,3%) receitas, seguida por Sinvastatina 20mg em 3158 (33,0%). Atorvastatina na dose de 40mg foi a terceira mais utilizada, em 1.087 (11,4%) receitas ambulatoriais. A Tabela 2 apresenta os níveis de CT e LDL-c de acordo com o medicamento e dose utilizados.

**Tabela 2:** Níveis séricos de colesterol total e sua fração LDL-c encontrados de acordo com o tipo e a dose da estatina. Pacientes com doses intermediárias não foram incluídos

Medicamento e dose diária	Número de pacientes	Colesterol total (mg/dL) Média ± DP	LDL-c (mg/dL) Média ± DP
Sinvastatina			
10mg	302	168 ± 43	96 ± 36
20mg	3.164	177 ± 49	103 ± 40
40mg	3.764	173 ± 52	100 ± 41
80mg	57	193 ± 66	110 ± 34
**Atorvastatina**			
10mg	92	184 ± 63	109 ± 53
20mg	481	176 ± 56	100 ± 42
40mg	1.088	170 ± 52	97 ± 41
80mg	283	182 ± 51	109 ± 43

DP: desvio padrão.

Verificamos que, no caso da sinvastatina, a dose crescente, aparentemente, foi utilizada em casos mais resistentes, sugerindo que uma adequação da dose foi realizada. Para aqueles em uso de atorvastatina, verificamos que o nível médio do CT e LDL-c decresce de modo não estatisticamente significante (p>0,05) com o incremento da dose até o nível de 40mg diários. A dose de 80 mg, utilizada em 3% dos indivíduos, foi possivelmente utilizada em indivíduos com menor resposta ao seu uso, pois ambos os valores de CT e LDL-c foram significativamente maiores que os dos que receberam atorvastatina 40mg diários (p<0,05). De modo geral, verifica-se que doses mais elevadas foram utilizadas, sugerindo a tentativa por um melhor controle da hipercolesterolemia.

Os níveis médios de CT e LDL-c foram menores significativamente (p<0,05) nos pacientes recebendo prescrições pela especialidade de cardiologia comparativamente aos outros grupos ambulatoriais, sendo o único com CT médio abaixo de 170mg/dL e LDL-c médio abaixo de 95mg/dL (Tabela 3).

**Tabela 3:** Níveis séricos médios de colesterol total e sua fração LDL-c de acordo com a especialidade prescritora no ano de 2016

Especialidade	Colesterol total (mg/dL)	LDL-c (mg/dL)
Cardiologia	166,1 ± 45,1	94,6 ± 36,3
Endocrinologia	179,5 ± 49,3	103,8 ± 40,3
Geriatria	171,6 ± 45,0	98,4 ± 37,2
Nefrologia	183,3 ± 58,5	104,6 ± 46,6
Neurologia	171,9 ± 45,1	104,5 ± 36,9
Vascular	175,0 ± 51,8	104,2 ± 39,4
Nutrologia	186,5 ± 47,0	112,7 ± 41,1
Outras	192,5 ± 53,4	115,3 ± 44,6

## Discussão

Neste estudo, pudemos verificar que a prescrição de estatinas em um hospital terciário do sistema público é bastante frequente, possivelmente pela densa concentração de pacientes com elevado número de comorbidades de ordem cardiovascular em seguimento na Instituição. Vannucchi et al., em nossa Instituição, nos anos 1970, descreveram o perfil lipídico em lipidogramas de cerca de 1.700 pacientes coletados durante período de cerca de 3 anos. Esses autores verificaram que 25,5% dos lipidogramas solicitados apresentavam alterações diagnósticas de pelo menos uma dislipidemia. Contudo, diante da inexistência de tratamentos específicos à época, nada foi discutido sobre o tópico da terapêutica.^7^ Na literatura mundial, Pant et al. reportaram dados de perfil lipídico em hospital de nível terciário no Nepal.^8^ Esses autores utilizaram uma amostra de conveniência ambulatorial sem registro do uso de estatinas ou comorbidades e verificaram que, em 408 casos avaliados com idade média de 50 anos, os valores médios de LDL-c e CT eram 113±41 mg/dL e 180±54mg/dL, respectivamente. Estudo na Turquia utilizou os resultados de lipidogramas para proceder à busca ativa de casos de HF, tendo verificado que muitos casos de elevação de LDL-c nem estavam recebendo tratamento.^9^

De modo original, nesta investigação, verificamos que a prescrição de estatinas ocorre de modo disseminado na Instituição. Tal fato indica o conhecimento do fator de risco cardiovascular, mas sem necessariamente visar a uma meta específica, pois há grande variabilidade nos valores médios em cada especialidade, notadamente naquelas lidando com doenças cardiovasculares (Divisão de Cirurgia Vascular do Departamento de Cirurgia e Departamento de Neurologia). Verificamos que a nutrologia, responsável por 1,1% das receitas emitidas, apresenta os níveis médios lipídicos mais elevados, fato possivelmente relacionado com a circunstância de que casos resistentes ou com HF são referenciados preferencialmente para esse ambulatório em nível terciário. Além disso, para essa amostra específica de pacientes, pode-se especular que, além de estatinas, outros medicamentos mais novos – como a ezetimiba, que reduz a absorção intestinal de colesterol, e os inibidores da proproteína convertase subtilisina/kexina tipo 9 (PCSK9) – ainda não estão disponíveis e devem ser necessários.

A utilização de atorvastatina foi modesta (22%). Por ser necessária a prescrição mais elaborada e demorada, com inclusão dos resultados laboratoriais, pode ter havido subutilização. O encontro de elevado percentual de pacientes em uso de estatinas sem que haja pelo menos um exame anual de controle sugere que não são utilizados protocolos clínicos locais ou diretrizes publicadas.^10^ Podemos ainda constatar que, em grande número de pacientes, nenhum ajuste medicamentoso é realizado, e a dose utilizada é prescrita de modo “automático”, ou seja, em critérios avalizados. Possivelmente, as doses em uso necessitariam ser ajustadas de modo periódico e coordenado, dentro de protocolos clínicos institucionais bem estabelecidos, visto que pacientes oriundos de ambulatórios vinculados fortemente com a ocorrência de doenças cardiovasculares, mas sem protocolos clínicos definidos, apresentam níveis médios significativamente maiores que aqueles observados nos ambulatórios de cardiologia, nos quais eles existem, sugerindo que o controle de fatores de risco é visto com enfoques distintos, ainda que inseridos em um mesmo contexto nosológico.

Apesar de os níveis médios globais estarem dentro de níveis aceitáveis para uma amostra da população geral, cabe lembrar que há um grande número de indivíduos com elevado risco cardiovascular nessa amostra, o que sugere a existência de espaço para maior redução dos valores médios. É possível ainda constatar uma elevada proporção de pacientes com valores muito elevados apesar da utilização de estatinas. Isso pode indicar um problema relativo à aderência ao tratamento. Como estão disponíveis gratuitamente na rede pública, não há por que discutir restrições financeiras, e, no período avaliado, não houve falta de medicamentos na rede assistencial.

Outro aspecto a ser ressaltado está no fato de que pacientes do gênero feminino apresentavam níveis lipídicos significativamente mais elevados que os do gênero masculino. A proporção de mulheres acima de 60 anos de idade com elevação do colesterol total é maior em várias séries,^11^^,^^12^ sem que uma clara explicação fosse oferecida. O papel da menopausa nesse incremento não pode ser desconsiderado. Embora especulativamente no contexto, é plausível supor que haja certa displicência com o ajuste medicamentoso em pacientes desse gênero no que tange ao controle de fatores de risco para doença arterial coronariana, algo também previamente relatado.^13^

O predomínio da utilização de sinvastatina possivelmente decorre da sua disponibilização pelo sistema único de saúde (SUS) em nível ambulatorial de forma mais disseminada. A utilização de atorvastatina depende de receituário especial, por se tratar de medicamento incluído no programa de alto custo do governo estadual, e tem sido reservada para casos refratários ou com intolerância à sinvastatina. De modo geral, verificamos a utilização em sua maioria com doses elevadas, seja de sinvastatina ou de atorvastatina.

A grande variabilidade nos níveis lipídicos associados às diversas doses utilizadas indica a necessidade de ajustes nas doses dos medicamentos e, possivelmente, na utilização de estatina com maior potência, reforçando ainda mais a necessidade de utilização de protocolos clínicos bem estabelecidos e de uso institucional para estabelecimento sobre a ação medicamentosa e as doses a serem utilizadas.

Por fim, o número de indivíduos com níveis de LDL-c acima de 190mg/dL, apesar do uso de estatinas, é significativamente mais elevado do que o reportado na população geral. Esse percentual (2,4%) reflete, muito provavelmente, como fator precípuo, a concentração em nível terciário de indivíduos com maior número de comorbidades. Entretanto, considerando ainda o fato de serem mais jovens, isso sugere fortemente a presença de HF, que deve ser investigada de modo sistematizado após se atingirem as doses máximas possíveis de estatina.

## Limitações

Diversas limitações estão presentes neste estudo. Em primeiro lugar, não incluímos todo o perfil lipídico, como os níveis de triglicerídios e colesterol da lipoproteína de alta densidade (HDL-c). Tal ocorrência deveu-se ao fato de ser artigo focado no uso de estatinas, e essas dosagens não interferirem diretamente na sua prescrição. Não foram coletados dados referentes ao uso de ezetimibe, que contribui para a redução dos lipídios mesmo em uso de doses elevadas de estatinas,^14^ pois não faz parte das medicações disponíveis no SUS, apesar de existirem prescrições desse medicamento no receituário ambulatorial, pois alguns pacientes conseguiam adquiri-lo. Outro aspecto limitante foi o fato de não terem sido coletadas as comorbidades e dados antropométricos apresentados pelos indivíduos. Infelizmente, em estudos como este, com grande número de dados, a revisão individual dos prontuários não é factível, e o sistema eletrônico de registro de dados (*big data*) ainda está em fase de implantação, e seu acesso nessa fase preliminar poderia gerar dados errôneos. Desse modo, uma classificação de risco cardiovascular dos pacientes não foi obtida.

## Conclusão

Nesta coorte transversal em um hospital terciário, foi possível verificar que a prescrição de estatinas é disseminada, mas que a obtenção de metas bem estabelecidas de controle de CT e LDL-c não é atingida em grande percentual de indivíduos, e que há, possivelmente, um elevado percentual de portadores de HF que necessitariam ser investigados apropriadamente para melhor definição diagnóstica e de enfoque terapêutico, por suas implicações prognósticas. A adesão institucional a protocolos clínicos diagnósticos e de tratamento mais uniformemente padronizado favoreceria melhor controle de dislipidemias em instituição terciária e a eventual alocação de recursos para prescrição de fármacos novos e efetivos, como os inibidores de PCSK9 em casos selecionados conforme diretrizes clínicas.^15^
