# Supplementary material for: Perfil de Prescrição de Estatinas e de Níveis Lipêmicos em Ambulatórios de Hospital Terciário Público
Source: Arq Bras Cardiol. 2021 Apr 8;116(4):736–41. [Article in Portuguese] doi: 10.36660/abc.20190513 (PMC8121392; doi:10.36660/abc.20190513)
Supplement: Translation

# Translation: Statins Prescriptions and Lipid Levels in a Tertiary Public Hospital

**Authors:** André Schmidt, Henrique Turin Moreira, Gustavo Jardim Volpe, Vamberto B. Foschini, Thiago Florentino Lascala, Minna Moreira Dias Romano, Marcus Vinícius Simões, José Ernesto dos Santos, Benedito Carlos Maciel, José Antonio Marin

**Affiliations:** 1 Ribeirão PretoSP Brazil Hospital das Clínicas da Faculdade de Medicina de Ribeirão Preto da Universidade de São Paulo (HCFMRP-USP), Ribeirão Preto, SP – Brazil

**Keywords:** Hydroxymethylglutaryl CO-Reductase Inhibitors, Dyipidemias, Hyperpoprotein Type II. Hypercholesterolemia Familial/therapy, Cholesterol, Public Hospital, Lipid Profile

## Abstract

**Background:**

The development of a new class of medications that are highly capable of reducing LDL-cholesterol renewed the interest in the characterization of familial hypercholesterolemia patients. Nevertheless, little is known about the lipid profile of patients in tertiary healthcare centers in Brazil in order to better estimate the real occurrence of familial hypercholesterolemia, with initial suspect of LDL-cholesterol levels above 190 mg/d/L.

**Objectives:**

This study evaluated the lipid profile (total cholesterol and LDL-cholesterol) in ambulatory patients from a general tertiary public hospital.

**Methods:**

Retrospective study comparing prescriptions of statins and lipid profile results. The significance level was established in 5%.

**Results:**

In one year, 9,594 individuals received statin prescriptions, of whom 51.5% were females and the mean age was 63.7±12.9 years-old (18 to 100 years-old). Thirty-two medical specialties prescribed statins. Cardiology was responsible for 43% of the total. Nearly 15% of those patients with a prescription did not have a recent total cholesterol result and 1,746 (18%) did not have a recent LDL-cholesterol measurement. The occurrence of the latter between 130 and 190 mg/dL was present in 1,643 (17.1%) individuals, and 228 (2.4%) patients had an LDL-cholesterol ≥190mg/dL among those using statins at distinct doses. Only two statins were used: simvastatin and atorvastatin. The first was prescribed in 77.6% of the prescriptions.

**Conclusion:**

In this cross-sectional cohort at a tertiary general hospital, statins have been widely prescribed but with little success in achieving recognized levels of control. There is probably a significant number of FH individuals in this cohort that need to be properly diagnosed in order to receive adequate treatment due to its prognostic implications.

## Introduction

Although a recent metanalysis indicates an incidence of 1:250 for familial hypercholesterolemia (FH) in the general population,^1^ the real specific prevalence of cases with high cholesterol values in tertiary public healthcare outpatient centers in Brazil is unknown. In general, these centers concentrate patients with more comorbidities and severe clinical presentations.

Only few studies have evaluated the cost-effectiveness of statins use in the Brazilian unified public health system (*Sistema Único de Saude* – SUS);^2^^,^^3^ however, adherence to treatment was evaluated in selected samples (women) and reached only 15.5% in a small series.^4^ The recent incorporation to the therapeutic arsenal of new highly effective medications for hypercholesterolemia control,^5^^,^^6^ although with high financial costs, led to a convergence of patients with a very ominous lipid profile to the public health system looking for the prescription of these medications. Nevertheless, little is known about the lipid profile and treatment of these outpatients in tertiary centers.

Our purpose is to report the real profile of statins prescription in a tertiary public hospital, profile of resultant lipids, and possible presence of FH patients (LDL-cholesterol > 190 mg/dL) despite the use of statins.

## Methods

This cross-sectional study was based on a systematic electronic data collection from the institution clinical records, including patients of both genders ≥ 18 years-old who received an ambulatory prescription of any statin, in the Hospital das Clínicas da Faculdade de Medicina de Ribeirão Preto da Universidade de São Paulo (HCFMRP-USP), which was a tertiary public teaching institution in 2016. In addition, total cholesterol (TC) and/or LDL-cholesterol (LDL-c) measurements were obtained later that year. For individuals with more than one measurement in such year, only the last one was considered. We collected information regarding gender, age, used statin and its dose, and the specialty that requested the prescription. Since an electronic search was performed in clinical records, no data were obtained about comorbidities and clinical and anthropometric parameters. This study was approved by the local institutional review board (CAAE 16516819.0.0000.5440).

### Statistical analysis

A descriptive analysis was carried out, and values were expressed as mean and standard deviation if a normal distribution by the Kolmogorov-Smirnov test was observed. Qualitative variables were expressed as percentages, and the Pearson's correlation test was used to correlate TC and LDL-c values. Unpaired Student's *t*-test was applied to compare age categories.

SPSS v.25 (IBM Corporation, EUA) was the statistical package used, and the level of significance was established as 5%.

## Results

### Prescriptions

In 2016, 9,594 patients followed at our institution received an ambulatory prescription of statins. A discrete larger number of women (51.5% - 4,942 patients) received a prescription of statins. Mean age was 63.7±12.9 years-old (18 to 100 years-old). A TC of 8,110 (84.8%) and LDL-c level of 7,848 (82.0%) were available, indicating that 1,484 (15.2%) patients received a prescription without a recent TC measurement, whereas 1,746 (18.0%) did not present an LDL-c measurement. All medical specialties had patients prescribed without a recent lipid profile, but nearly 75% of them had a statin prescribed without a LDL-c recent result in Vascular Surgery.

Among the 32 medical specialties that prescribed statins, Cardiology was responsible for 43.5%, followed by Vascular Surgery (9.2%) and Nephrology (8.6%). The remaining 32% was distributed among the other medical specialties of this public tertiary hospital, and Nutrology was responsible for only 106 (1.1%), as seen in Table 1.

**Table 1:** Prescription of statins according to medical specialty in the year of 2016

Specialty	Number (%)
Cardiology	4160 (43.5)
Vascular surgery	1576 (9.2)
Nephrology	819 (8.6)
Neurology	731 (7.6)
Geriatrics	657 (6.9)
Endocrinology	653 (6.8)
Nutrology	94 (1.0)
Other 25 specialties	1,576 (16.5)
Total	9,567 (100)

### Lipid profile

The mean TC of four samples was 174.4± 49.5 mg/dL (40.0–739.0 mg/dL) and mean LDL-c was 101.1±40.0 mg/dL (4.0–635.0 mg/dL). A strong correlation between these two variables was observed (r= 0.94 - p<0.001). Figure 1 presents the individual values for TC and LDL-c.

**Figure 1:** Histograms of the absolute values of total cholesterol (A) and low-density cholesterol fraction – LDL-c; (B) in the sample of patients from Hospital das Clínicas, Faculdade de Medicina de Ribeirão Preto, year 2016

Women had significantly worse values for TC (183.3±49.9 *versus* 164.5±47.0 mg/dL; p<0.001) and LDL-c (107.1±40.9 *versus* 94.3±7.9 mg/dL; p<0.001) than men, although the mean age was equal: 63.65±13.56 *versus* 63.36±12.60; p=0.29.

LDL-c levels above 130 mg/dL and below 190 mg/dL were observed in 1,643 (17.1%) patients with a prescription of statins. In addition, 18.2% of the total sample did not have an LDL-c measurement despite the prescription of a statin. Therefore, a considerable number of patients presented an LDL-c value above recommendations of guidelines, despite using statins and without taking other comorbidities into account.

Finally, 228 (2.4%) patients presented LDL-c ≥ 190 mg/dL and were prescribed distinct statins in various dosages. Two-thirds (152) were females and had a mean age below the whole sample (55±15 *versus* 63±13years-old; p<0.05), which is a possible indicator of the occurrence of FH in this tertiary hospital group of patients.

### Statin use

Since this is a hospital part of the Brazilian public health system, only two statins were available for prescription: Simvastatin e Atorvastatin. The first was 77.6% (7,474) of the recipes. Simvastatin 40 mg was the most used dosage in 3,760 (39.3%) prescriptions, followed by its dosage of 20 mg in 3,158 (33.0%). Atorvastatin 40 mg was the third most used in 1,087 (11.4%) ambulatory prescriptions. Table 2 summarizes TC and LDL-c levels according to the type of statin and dosage in the prescription.

**Table 2:** Mean serum levels of total cholesterol and its low-density cholesterol fraction according to statin used and its daily dosage. Patients using intermediary dosages were excluded

Statin and daily dosage	Number of patients	Total cholesterol (mg/dL) mean±SD	LDL-c (mg/dL) mean±SD
Simvastatin			
10 mg	302	168±43	96±36
20 mg	3164	177±49	103±40
40 mg	3764	173±52	100±41
80 mg	57	193±66	110±34
**Atorvastatin**			
10 mg	92	184±63	109±53
20 mg	481	176±56	100±42
40 mg	1088	170±52	97±41
80 mg	283	182±51	109±43

SD: standard deviation.

We verified that higher simvastatin dosages were probably delivered in patients with the worst lipid profile, indicating that dosage adjustments were being applied. Both TC and LDL-c levels reduced without statistical significance (p>0.05) for atorvastatin until a daily dosage of 40 mg was achieved. The 80-mg dosage was prescribed to only 3% of our sample. Those, individuals had a worse response, since both TC and LDL-c levels were higher than in those who received atorvastatin 40 mg daily (p<0.05). In general, higher dosages were prescribed that suggest a better lipid level control.

Mean TC and LDL-c levels were significantly (p<0.05) lower in patients prescribed by Cardiology in comparison to other specialties, which is the only specialty with a mean TC below 170 mg/dL and mean LDL-c below 95 mg/dL (Table 3).

**Table 3:** Mean serum levels of total cholesterol and its low-density cholesterol fraction (LDL-c) according to medical specialty in the year 2016

Specialty	Total cholesterol (mg/dL)	LDL-c (mg/dL)
Cardiology	166.1±45.1	94.6±36.3
Vascular surgery	179.5±49.3	103.8±40.3
Nephrology	171.6±45.0	98.4±37.2
Neurology	183.3±58.5	104.6±46.6
Geriatrics	171.9±45.1	104.5±36.9
Endocrinology	175.0±51.8	104.2±39.4
Nutrology	186.5±47.0	112.7±41.1
Others	192.5±53.4	115.3±44.6

## Discussion

In this study we verified that prescribing statins is common practice in a tertiary public hospital environment, probably related to the elevated presence number of cardiovascular comorbidities present in this selected sample. Vannucchi et al., in our institution in the late 1970s, described the lipid profile of nearly 1,700 patients obtained during a three-year period. They verified that 25.5% of the exams presented at least one diagnosis of dyslipidemia. Yet, due to the absence of specific treatments back then, nothing was discussed about this topic.^7^ In the literature, Pant et al. reported the lipid profile of a tertiary hospital in Nepal.^8^ This study obtained a convenience outpatient sample of patients that did not use statins or had comorbidities and verified that in 408 patients with a mean age of 50, LDL-c and TC were 113±41 mg/dL and 180±54 mg/dL, respectively. Another study from Turkey used the lipid profile results to identify FH patients and concluded that many patients with elevated LDL-c were not receiving any specific treatment.^9^

As an original contribution of our investigation, we reported that prescription of statins is disseminated at our institution. This indicates a widespread of this cardiovascular risk factor, apparently without a specific target, since the great variability of the mean values for each specialty is notably those dealing directly with cardiovascular diseases (vascular surgery division in the Surgery Department and the Neurology Department). The fact that the Nutrology Division, responsible for 1.1% of the prescriptions, presented the highest lipid levels probably indicates that resistant patients or those with FH are probably referred to their tertiary level outpatient clinic. In addition, this specific sample suggests that new medications, such as ezetimibe, which reduce the intestinal absorption of cholesterol and the proprotein convertase subtilisin/kexin type 9 (*PCSK9*), although not available for prescription in the public health system, may be needed.

Atorvastatin prescription was modest (22%), considering that an elaborated procedure for statin prescription with the inclusion of laboratorial results and underutilization of this statin may have occurred. The elevated percentage of patients receiving statins without at least one annual lipid measurement suggests that local clinical protocols or societies guidelines were not observed.^10^ Additionally, in a great number of patients, no dosage adjustments were performed, and the prescription dosage was automatically repeated.

Periodical dosage adjustments guided by well-established institutional clinical protocols should have been applied. Patients from services that are highly associated with the occurrence of cardiovascular diseases without well-established treatment protocols presented higher mean lipid levels than those observed in Cardiology ambulatories where they exist, suggesting that the enforcement of risk factors control is distinct despite being in the same nosological context.

Although the mean lipid levels of our sample were within those acceptable for a general population, the large number of individuals with elevated cardiovascular risk in our sample suggests the existence of space for improvement. In addition, there is a significant proportion of patients with very high lipid levels despite the use of statins. It may indicate an adherence to treatment problem. Since these statins are distributed without charge by the public health system, there is no point in considering financial restrictions and, for the study period, no lack of medication in the public pharmacies was reported.

Another relevant aspect to be considered is that female patients had higher lipid levels compared to male patients. The proportion of women older than 60 years-old was reported in other studies^11^^,^^12^ without any clear reason. The role of menopause in these increased values needs to be considered. Although speculative in this context, it is plausible to attribute some lenience with dosage adjustments in this gender for controlling coronary artery disease risk factors, as previously reported.^13^

The preponderant prescription of simvastatin is probably related to its widespread availability in the ambulatory public health system. Atorvastatin needs a special request since it is included in a program to deliver high-cost medications of the São Paulo state government and preferred in refractory patients or in simvastatin intolerance. In general, we observed a high dosage use of both statins.

The high variability in lipid levels related to the various dosages indicates that adjustments and use of a more effective statin is a necessity, reinforcing the need for well-established institutional clinical protocols to improve drug and dosage selection.

Finally, the number of individuals with LDL-c above 190 mg/dL, despite the use of statins, is significantly higher than the one reported in the general population. This percentage (2.4%) certainly reflects the high concentration of patients, in this tertiary level, with more comorbidities. Nevertheless, since these patients were younger, the occurrence of FH is a strong possibility that needs systematic investigation after reaching the higher statins dosages.

## Limitations

Our study presents many limitations. First, we did not include the complete lipid profile, with triglycerides and high-density cholesterol (HDL-c) levels, because our focus was on the use of statins, and their levels do not interfere with statin prescription. No data on the use of ezetimibe were collected, even though its use may have contributed to the reduction of lipids,^14^ because it was not included in the list of medications available in the Brazilian public health system, although some patients may have acquired it by suggestion of their physicians. Another limitation is the fact that comorbidities and anthropometric data were not obtained. Unfortunately, in studies like this, with a large number of patients, revision of patient's notes individually is not feasible and as the big data system is being established at the moment of the data collection of our study, many errors could have occurred. For this reason, there is no cardiovascular risk numbers.

## Conclusions

In this cross-sectional cohort of a tertiary hospital, we observed that prescription of statins is widespread, but clear TC and LDL-c targets are not achieved in a high proportion of patients. It is possible that a high percentage of FH exists and should be better investigated, for prognostic reasons. Institutional adherence to uniform clinical diagnostic and treatment protocols would probably increase a better control of dyslipidemia in this tertiary institution. It would also allow resource allocation for prescription of new effective medications, such as PCSK9 inhibitors in selected patients as recommended by guidelines.^15^
